# Electronically-Controlled Beam-Steering through Vanadium Dioxide Metasurfaces

**DOI:** 10.1038/srep35439

**Published:** 2016-10-14

**Authors:** Mohammed Reza M. Hashemi, Shang-Hua Yang, Tongyu Wang, Nelson Sepúlveda, Mona Jarrahi

**Affiliations:** 1 Electrical Engineering Department, University of California Los Angeles, USA; 2 Electrical Engineering and Computer Science Department, University of Michigan Ann Arbor, USA; 3 Electrical and Computer Engineering Department, Michigan State University, USA

## Abstract

Engineered metamaterials offer unique functionalities for manipulating the spectral and spatial properties of electromagnetic waves in unconventional ways. Here, we report a novel approach for making reconfigurable metasurfaces capable of deflecting electromagnetic waves in an electronically controllable fashion. This is accomplished by tilting the phase front of waves through a two-dimensional array of resonant metasurface unit-cells with electronically-controlled phase-change materials embedded inside. Such metasurfaces can be placed at the output facet of any electromagnetic radiation source to deflect electromagnetic waves at a desired frequency, ranging from millimeter-wave to far-infrared frequencies. Our design does not use any mechanical elements, external light sources, or reflectarrays, creating, for the first time, a highly robust and fully-integrated beam-steering device solution. We demonstrate a proof-of-concept beam-steering metasurface optimized for operation at 100 GHz, offering up to 44° beam deflection in both horizontal and vertical directions. Dynamic control of electromagnetic wave propagation direction through this unique platform could be transformative for various imaging, sensing, and communication applications, among others.

Electronically-controlled beam-steering techniques based on phased array antennas and leaky wave antennas are very successful in the radio frequency regime^1^,^2^,^3^. However, they lose efficiency at higher frequencies due to the reduction in signal gains and increase in signal losses associated with electronic circuits. Instead, mechanical beam-steering techniques are traditionally used in the terahertz and optical frequencies. Despite extensive progress in mechanical beam-steering techniques through miniaturization and utilization of Micro Electro Mechanical Systems (MEMS), their scope of the potential use is still limited by their high complexity, alignment sensitivity, low reliability, and low speeds^4^,^5^,^6^,^7^,^8^. Non-mechanical techniques have been introduced as an alternative, which achieve electromagnetic beam-steering by introducing a phase gradient along the beam path. However, all the proposed approaches for introducing a variable phase gradient have utilized either an external light source and spatial light modulator or reflectarrays^9^,^10^,^11^,^12^, which cannot be used in many practical applications due to their complex packaging and tight alignment requirements. To address these limitations, we present an electronically-controlled metasurface that can be placed at the output facet of any millimeter-wave/terahertz/far-infrared electromagnetic radiation source to control the direction of the generated beam (Fig. 1). It consists of a two-dimensional array of resonant metasurface unit-cells fabricated on a substrate with electronically-tunable dielectric constant. By controlling the applied current to each individual metasurface unit-cell, its resonance frequency is adjusted and the phase of the transmitted electromagnetic wave through the unit-cell is controlled accordingly. This allows manipulating the phase front of the transmitted electromagnetic wave through the metasurface for various beam-steering and beam-shaping purposes.

The described beam-steering metasurface concept can be designed for operation at a desirable millimeter-wave/terahertz/far-infrared frequency by an appropriate choice of metasurface geometry. In this work, we present a design optimized for operation at 100 GHz frequency. For this implementation, vanadium dioxide (VO_2_) is used as the substrate with electronically-tunable dielectric constant. VO_2_ is a correlated electron material, which has semiconductor-like behavior at room temperature but goes through an abrupt insulator-to-metal transition triggered by thermal, electrical, optical, or mechanical stimuli, introducing a significant change in its dielectric, mechanical, and optical properties^13^,^14^,^15^,^16^. This solid-to-solid phase transformation process is completely reversible and, being a first-order transformation, shows a hysteresis behavior. In the case of thermal triggering, for example, the insulator-to-metal transition occurs at ~68° resulting in a refractive index variation from 2.6 to 36 and an extinction coefficient variation from near 0 to 16 at 100 GHz for a 200 nm thick VO_2_ layer grown on a lightly doped Si substrate between 55 °C and 90 °C^14^.

Schematic diagram and scanning electron microscope (SEM) image of a fabricated beam-steering metasurface is shown in Fig. 2a. It consists of a two-dimensional array of cross-shape apertures in a 200 nm thick Au layer placed on a 200 nm thick VO_2_ layer grown on a lightly doped Si substrate. Each metasurface unit-cell consists of a resistive heater electrode placed at the perimeter of the cross-shape aperture on top of the VO_2_ layer for Joule heating actuation. The resistive heater electrodes are electrically insulated from the top Au layer through a 100 nm-thick SiO_2_ layer. The top Au layer shadows the heater electrodes from the incident electromagnetic wave. This offers a flexible platform for the actuation of metasurface unit-cells, without impacting the electromagnetic properties of the metasurface. The applied current to the heating electrode of each metasurface unit-cell controls its resonance frequency and the phase shift of a transmitted electromagnetic wave^17^,^18^. By controlling the applied current to each individual metasurface unit-cell, the phase front of the transmitted electromagnetic wave through the metasurface is varied and the transmitted beam is deflected to the specified direction.

The width and the height of each heating electrode are 5.0 μm and 50 nm, respectively. Each metasurface unit-cell is actuated by an independent heating electrode that is routed through the metasurface while maintaining a large distance from the cross-shape aperture of other unit-cells. The heating electrodes are designed to vary the temperature of the VO_2_ layer locally underneath the cross-shape apertures of each individual unit-cell independently to trigger the insulator-to-metal transition without impacting the other unit-cells (Fig. 2b). A multi-physics finite-element solver (COMSOL) is used to calculate the temperature profile of the VO_2_ layer as a function of the applied current to the heating electrode of each unit-cell. As shown in Fig. 2b inset, a temperature variation of 72–74 °C is expected in the VO_2_ layer underneath the cross-shape apertures at an applied current of 13 mA to all the metasurface unit-cells, which is sufficient for an insulator-to-metal transition.

The geometry of the cross-shape apertures (817.0 μm periodicity in each direction and 567 × 35 μm^2^ size on each arm) is chosen to offer a polarization-independent resonant transmission for a 100 GHz electromagnetic wave at room temperature. The resonance frequency of the metasurface unit-cells is shifted when passing a current through their heating electrode. This is due to refractive index variations in the VO_2_ layer, introducing a phase shift in a transmitted electromagnetic wave at 100 GHz. A finite-element-based full-wave electromagnetic solver (ANSYS HFSS) is used to calculate the amplitude and phase of the transmission spectrum through the metasurface unit-cell as a function of the applied current to the heating electrode (Fig. 2c). For this purpose, the calculated temperature profile of the VO_2_ layer is used to determine the refractive index and extinction coefficient profile inside the VO_2_ layer^14^. The results indicate an electronically-controlled phase shift for a transmitted wave through the metasurface unit-cell at 100 GHz. An 8 GHz shift in the metasurface resonance frequency is estimated when applying a 13 mA current, resulting in 59° phase shift and 50% intensity modulation for a transmitted wave at 100 GHz. Figure 2c inset shows the color map transmission profile of a 100 GHz electromagnetic wave incident on a metasurface unit-cell for two orthogonal polarizations along the cross shape aperture axis. As expected from the design symmetry, both polarizations offer the same overlap between the temperature profile and electromagnetic transmission profile of the metasurface unit-cell, indicating polarization-independent phase/intensity modulation behavior for the designed metasurface.

## Results

**Figure 2:**
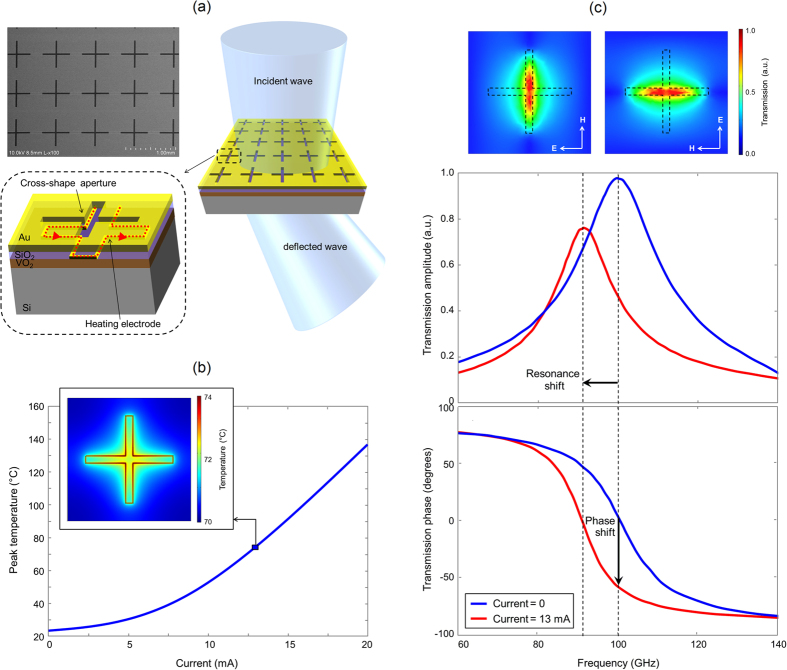
Operation principles of the electronically-controlled beam-steering metasurface. (**a**) Schematic diagram and top-view scanning electron microscope image of a prototype beam-steering metasurface designed for operation at 100 GHz. The applied current to the heating electrode of each metasurface unit-cell (red arrow) controls its resonance frequency and the phase shift of a transmitted electromagnetic wave through the metasurface unit-cell. By controlling the applied current to each individual metasurface unit-cell, the phase front of the transmitted electromagnetic wave through the metasurface is varied and the transmitted beam is deflected to the specified direction. (**b**) Temperature of the VO_2_ layer as a function of the applied current to the heating electrode. Inset shows the color map temperature profile of the VO_2_ layer at an applied current of 13 mA to all the metasurface unit-cells. (**c**) Amplitude and phase of the metasurface transmission spectrum, indicating a polarization-independent resonant transmission at 100 GHz at room temperature. The resonant transmission peak is shifted to 92 GHz at an applied current of 13 mA, resulting in 59° phase shift and 50% intensity modulation for a transmitted wave at 100 GHz.

The beam-steering metasurface prototype is fabricated on a 200 nm-thick VO_2_ film deposited on a 500 μm-thick lightly doped Si substrate. First, the VO_2_ film is deposited by a pulsed laser deposition system, as described in the methods section. Characteristics of the deposited VO_2_ film are shown in Supplementary Fig. S1. Next, the heating electrodes are formed by optical lithography followed by Ti/Au (5/45 nm) deposition and liftoff. Then, the 100 nm-thick SiO_2_ insulating layer is deposited using electron beam evaporation. Finally, the cross-shape apertures are formed by optical lithography followed by Ti/Au (10/100 nm) deposition and liftoff. During the entire fabrication process after the VO_2_ deposition the temperature of the sample is kept below 90 °C. An SEM image of the fabricated metasurface prototype is shown in Fig. 2a.

**Figure 3:**
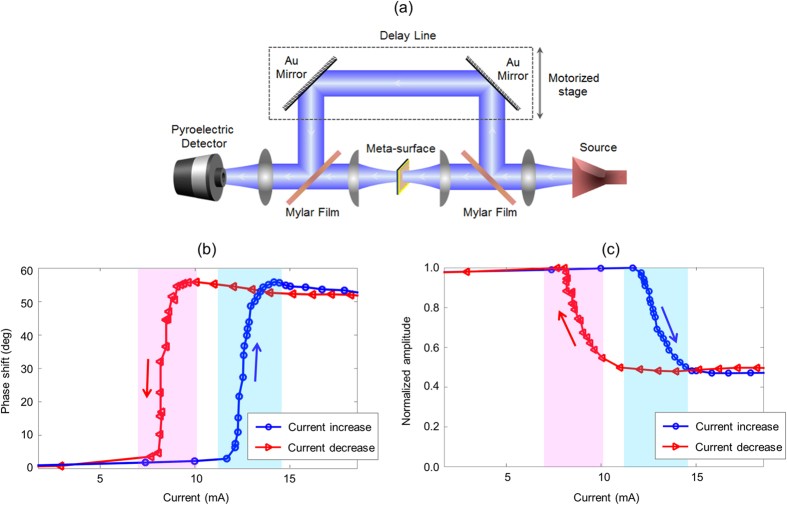
Phase and intensity modulation characteristics of the fabricated metasurface. (**a**) Block diagram of the frequency-domain interferometry setup used for characterizing the phase/intensity modulation behavior of the fabricated metasurface. The measured phase response and amplitude response of the fabricated metasurface at 100 GHz as a function of the applied current are shown in (**b,c**), respectively.

The phase/intensity modulation behavior of the fabricated metasurface are characterized in a frequency-domain interferometry setup (Fig. 3a) when applying identical currents to the heating electrodes of the metasurface unit-cells. The interferometry setup consists of a frequency-tunable millimeter-wave source (Millitech AMC-10RFHB0 ×6 multiplier) connected to a WR-10 horn antenna. The radiated beam is collimated by a biconvex polyethylene lens and split into two beams by a Mylar film. One beam is focused onto the metasurface by a plano-convex Teflon lens and the other beam is delayed by a motorized delay stage. The transmitted beam through the metasurface is collimated by another plano-convex Teflon lens and combined with the delayed beam by a Mylar film. The combined beam is focused onto a pyroelectric detector by a biconvex polyethylene lens for power measurements. The output power of the interferometer, which is the result of interference between the transmitted beam through the metasurface and the time-delayed beam, is measured as a function of the delay path length. The phase and intensity modulation introduced by the metasurface are calculated from the value and location of the interference pattern peaks at different current levels applied to the heating electrodes.

The introduced phase shift and intensity modulation by the metasurface at 100 GHz are shown in Fig. 3b,c, respectively. The results indicate an abrupt insulator-to-metal transition in VO_2_ when increasing the applied current from 12 mA to 14 mA (blue curves), implying a peak temperature increase from 55 °C to 90 °C in this range. Up to 57° phase shift is observed at 14 mA accompanied by a 50% amplitude modulation. This amplitude modulation is associated with the shift in the metasurface resonance frequency as well as the increase in the conductivity of the VO_2_ layer. Similar intensity modulation levels and phase shifts ranging from 55° to 60° are observed within 80–100 GHz frequency range [Supplementary Fig. S2]. In order to investigate the impact of the hysteresis in VO_2_ characteristics, the introduced phase shift and intensity modulation by the metasurface are measured when reducing the applied current. The results indicate an abrupt metal-to-insulator transition in VO_2_ when decreasing the applied current from 10 mA to 8 mA (red curves). This hysteresis should be taken into account for adjusting the applied currents to the metasurface unit-cells when using the device for various beam-shaping and beam-steering applications.

The relatively linear phase shift behavior of the fabricated metasurface at the applied current range of 12–14 mA is used to adjust the device parameters for beam-steering at 100 GHz. For this purpose, the applied current to the metasurface unit-cells is linearly increased in the desired beam-steering direction to tilt the phase front of the transmitted beam through the metasurface. The beam deflection angle can be predicted using the generalized Snell’s law of refraction as 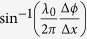, where *λ*_0_ is the vacuum wavelength and 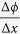 is the phase gradient in the desired direction for beam-steering, *x*. The fabricated metasurface consists of a 4 × 4 array of metasurface unit-cells with a 817 μm spacing in the horizontal and vertical directions. Therefore, beam deflection angles as large as ± 12° are predicted to be achieved in the horizontal and vertical directions when introducing phase gradients as large as ± 23°/mm by linearly distributing ± 57° phase change along 4 metasurface rows. However, the nonlinear phase modulation characteristics of the metasurface introduce larger phase gradients and beam deflection angles when linearly increasing the applied current in the desired beam-steering direction.

**Figure 4:**
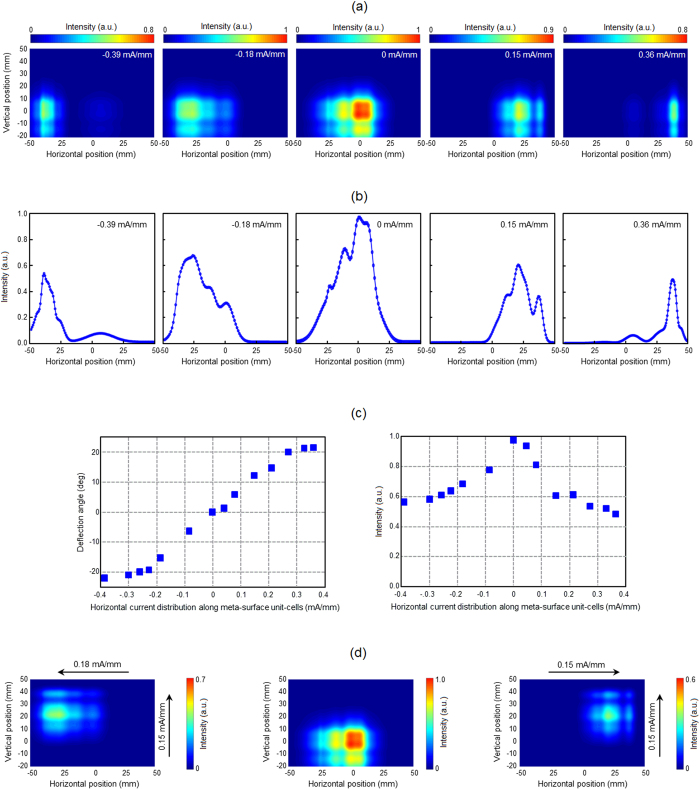
Performance of the fabricated beam-steering metasurface. (**a**) The color map of the intensity profile of the transmitted beam through the fabricated metasurface for various current distributions applied to the metasurface unit-cells in the horizontal direction, while keeping a uniform current distribution in the vertical direction. The middle color plot shows the beam profile when a uniform current distribution (12 mA) is applied to all the metasurface unit-cells in the horizontal and vertical directions. (**b**) The intensity profile of the transmitted beam at zero vertical elevation relative to the incident beam on the metasurface for various current distributions applied to the metasurface unit-cells in the horizontal direction, while keeping a uniform current distribution in the vertical direction. (**c**) The beam deflection angle (left) and beam intensity (right) as a function of the applied current distribution to the metasurface unit-cells. (**d**) The color map of the intensity profile of the transmitted beam for various current distributions applied to the metasurface unit-cells in the horizontal and vertical directions. The middle color plot shows the beam profile when a uniform current distribution (12 mA) is applied to all the metasurface unit-cells in the horizontal and vertical directions. The left color plot shows the beam profile when a current distribution of −0.18 mA/mm in the horizontal direction and 0.15 mA/mm in the vertical direction is applied to the metasurface unit-cells. The right color plot shows the beam profile when a current distribution of 0.15 mA/mm in the horizontal direction and 0.15 mA/mm in the vertical direction is applied to the metasurface unit-cells.

The beam-steering characteristics of the fabricated metasurface are monitored by a pyroelectric detector mounted on a motorized XYZ translation stage, scanning the beam intensity in a plane 100 mm away from the metasurface plane. For these measurements, the position of the 100 GHz source (Millitech AMC-10RFHB0 ×6 multiplier) is fixed relative to the metasurface. Figure 4a shows the color map of the projected intensity profile of the transmitted beam for various current distributions applied to the metasurface unit-cells in the horizontal direction, while keeping a uniform current distribution in the vertical direction. While no beam deflection is observed in the vertical direction, the position of the transmitted beam is shifted by −40 mm, −25 mm, 21 mm, and 40 mm in the horizontal direction when the applied current is swept horizontally (left-to-right). Using the center of the array as the spatial and angular origin, applied currents of −0.39 mA/mm, −0.18 mA/mm, 0.15 mA/mm, and 0.36 mA/mm produce beam deflection angles of −22°, −14°, 12°, and 22°, respectively. Note that the negative signs the current distribution values represent an increase in the current value from right to left. Figure 4b shows the intensity profile of the transmitted beam at zero vertical elevation relative to the incident beam on the metasurface. As expected, the beam deflection is accompanied by a reduction in the beam intensity due to the inherent tradeoff between the phase and intensity modulation introduced by the utilized phase modulation mechanism. Figure 4c shows the tradeoff between the beam deflection angle and attenuation of the fabricated beam-steering metasurface at 100 GHz. A total beam deflection angle of 44° is achieved in the horizontal direction when varying the applied current distribution in the horizontal direction from −0.39 mA/mm to 0.36 mA/mm.

Figure 4d shows the color map of the projected intensity profile of the transmitted beam for various current distributions applied to the metasurface unit-cells in the horizontal and vertical directions, deflecting the transmitted beam through the metasurface in both horizontal and vertical directions. When a current distribution of −0.18 mA/mm in the horizontal direction and 0.15 mA/mm in the vertical direction is applied to the metasurface unit-cells, the beam is deflected by −14° and 12° in the horizontal and vertical directions, respectively. Similarly, when a current distribution of 0.15 mA/mm in the horizontal direction and 0.15 mA/mm in the vertical direction is applied to the metasurface unit-cells, the beam is deflected by 12° and 12° in the horizontal and vertical directions, respectively. Since Joule heating is used as the stimuli triggering insulator-to-metal transition in VO_2_ and the unit-cells have a relatively large thermal mass, the beam-steering speeds offered by the proof-of-concept metasurface are limited to millisecond time-scales. However, it should be noted that much faster speeds can be offered when utilizing an electric field stimuli for triggering insulator-to-metal transition in VO_2_^19^,^20^,^21^,^22^.

## Discussion

The controllable properties of artificially structured metamaterials have offered unprecedented functionalities for manipulating spectral and spatial properties of electromagnetic waves^23^ and have enabled new electromagnetic phenomena such as negative index of refraction, perfect lensing, cloaking, etc^24^,^25^,^26^,^27^,^28^. The presented reconfigurable metasurface concept extends these unique capabilities to dynamic electromagnetic beam-steering and beam-shaping through an electrically-controlled and fully-integrated device platform, for the first time. Unlike previously suggested schemes, the presented reconfigurable metasurface concept does not require any external light source or a reflectarray element, making it suitable for direct integration with a variety of electromagnetic radiation sources such as photoconductive terahertz sources, solid-state waveguide lasers, quantum-cascade lasers, vertical external cavity surface emitting lasers (VECSELs). In fact, the reconfigurable metasurface layer can be implemented on any type of substrate with a refractive index matching the electromagnetic source substrate, eliminating the need for external lenses or corrugations patterned at the laser facet for enhancing beam coupling and/or beam-shaping purposes^29^,^30^. While the proof-of-concept metasurface demonstrated in this work is designed for operation at 100 GHz, the presented concept is universal and can be utilized for dynamic electromagnetic beam-steering and beam-shaping at other frequencies ranging from millimeter-wave to far-infrared frequencies. This is because the spectral response of the presented reconfigurable metasurface can be controlled by the geometry of the sub-wavelength metasurface unit-cells and their spacing, thus, is not limited by the characteristics of naturally existing materials. In fact, significantly larger beam deflection angles are expected to be offered by the presented metasurface concept when scaled in size to operate at optical frequencies. This is because of a much closer spacing between the phase-shifting metasurface unit-cells when miniaturized to operate at higher frequencies, leading to a much larger phase gradient in the desired beam-steering direction. Moreover, the two-dimensional symmetry of the presented metasurface offers a polarization-insensitive device operation, making it suitable for integration with electromagnetic radiation sources with arbitrary polarization.

The presented reconfigurable metasurface concept is not limited to VO_2_-based devices and alternative phase change materials such as liquid crystals, graphene, and reverse-biased Schottky junctions can be incorporated in the active region of the metasurface unit-cells to electronically tune the material refractive index and metasurface resonance frequency^31^,^32^,^33^. As such, much faster beam-deflection speeds can be offered by the presented reconfigurable metasurface concept if needed. Moreover, electronic control of the phase front of electromagnetic radiation through the presented reconfigurable metasurface concept can be utilized for other applications such as manipulating polarization of electromagnetic waves and holography^34^,^35^,^36^,^37^,^38^,^39^.

## Methods

Vanadium Dioxide (VO_2_) Deposition Procedure: The undoped VO_2_ thin film is deposited by pulsed laser deposition on a two-inch Si wafer. After reaching a background pressure of ~10^−6^ Torr, ultra-high purity (99.993%) oxygen is introduced to the chamber and a closed-loop regulated valve is used to maintain the pressure inside the chamber at 15 mTorr. On the back of the sample, a ceramic heater is maintained at 595 °C, which is translated to a temperature of approximately 470 °C at the sample. In the meantime, a KrF excimer laser (LambdaPhysik LPX 200, λ = 248 nm) at 10 Hz repetition rate and 350 mJ pulse energy (fluence ~2 J/cm^2^) is incident on a rotating vanadium target for 25 min. A customized and in-house built substrate holder maintains a uniform temperature distribution on the sample. The complete coverage of the plume on the substrate and the small temperature gradient across the sample during deposition allows a uniform VO_2_ film thickness across the two-inch Si substrate. After VO_2_ deposition, a 30 minute post-annealing step is performed at the same deposition environment conditions and temperature. VO_2_ thin films deposited by the described procedure typically result in highly oriented films with the monoclinic (011)_M1_ and rutile (110)_R_ planes parallel to the sample surface for room temperature and after phase transition^40^,^41^,^42^. The film thickness is measured using a profilometer at different locations on the wafer and found to be 200 ± 10 nm. The surface roughness of the VO_2_ thin film is measured at different locations on the sample, indicating a RMS surface roughness of 5 nm. The polycrystalline nature of the deposited VO_2_ film is observed by using scanning electron microscopy, which shows crystallites with average size of ~100 nm [Supplementary Fig. S1].

## Additional Information

**How to cite this article**: Hashemi, M. R. M. *et al*. Electronically-Controlled Beam-Steering through Vanadium Dioxide Metasurfaces. *Sci. Rep.* **6**, 35439; doi: 10.1038/srep35439 (2016).

## Supplementary Material

Supplementary Information

## Figures and Tables

**Figure 1:**
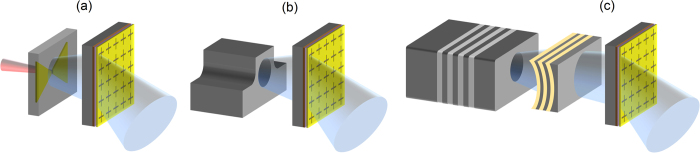
Integration compatibility of the presented beam-steering metasurface. The presented metasurface concept that can be placed at the output facet of any millimeter-wave/terahertz/far-infrared electromagnetic radiation source such as a photoconductive terahertz source (**a**), a solid-state waveguide laser (**b**), and a vertical external cavity surface emitting laser (VECSEL) (**c**) to control the direction of the generated beam.
