# Supplementary material for: Electronically-Controlled Beam-Steering through Vanadium Dioxide Metasurfaces
Source: Sci Rep. 2016 Oct 14;6:35439. doi: 10.1038/srep35439 (PMC5064393; doi:10.1038/srep35439)
Supplement: Supplementary Information [file srep35439-s1.pdf]

Electronically-Controlled Beam-Steering through Vanadium Dioxide Metasurfaces
Supplementary Information

Mohammed Reza M. Hashemi [(1)], Shang-Hua Yang [(1,2)], Tongyu Wang [(3)], Nelson Sepúlveda [(3)], and Mona Jarrahi [(1,2)]

[(1)] Electrical Engineering Department, University of California Los Angeles
[(2)] Electrical Engineering and Computer Science Department, University of Michigan Ann Arbor
[(3)] Electrical Engineering and Computer Science Department. Michigan State University

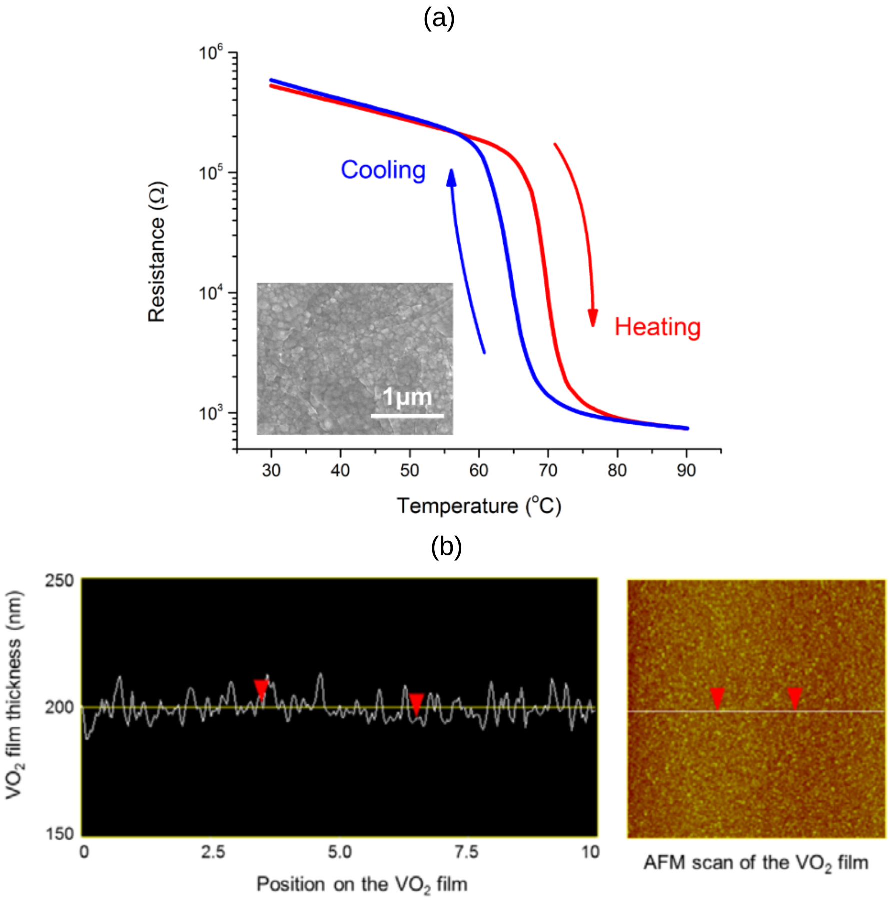

Supplementary Figure S1. Characteristics of the deposited VO_2 thin films: (a) Resistance of the deposited VO_2 thin film drops as the temperature is increased above the transition temperature of VO_2 (~ 68 °C). A temperature controller (Thorlabs, TED4015; which has temperature stability and resolution of 0.002 °C) is used to monitor and control the temperature of a Peltier heater attached to the bottom of the sample during the measurement. The hysteretic behavior across the phase transition and the drop in resistance (close to 3 orders of magnitude drop from 30 °C to 90 °C) show that the VO_2 thin film is highly stoichiometric. Inset shows the scanning electron microscope (SEM) image of the deposited VO_2 film, indicating polycrystalline nature of the deposited film and crystallites with average size of ~100 nm. (b) The surface roughness of the VO_2 thin film measured at different locations on the sample using atomic force microscopy (AFM), showing a RMS surface roughness of 5 nm (~2.5% of the film's thickness) over a 10 µm window size.

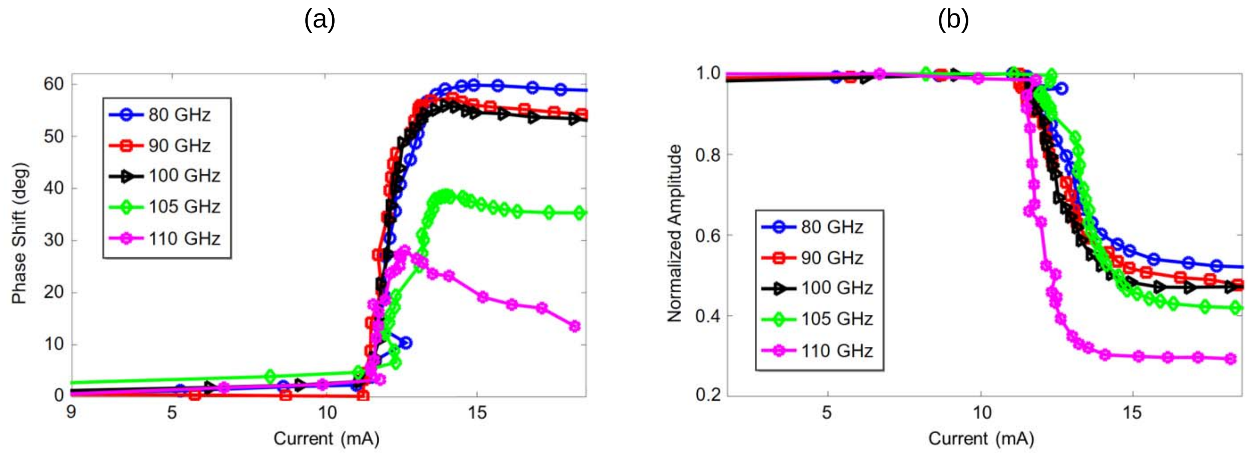

Supplementary Figure S2. Phase and intensity modulation characteristics of the fabricated metasurface as a function of frequency. The measured phase response and amplitude response of the fabricated metasurface at frequencies ranging from 80 GHz to 100 GHz as a function of the applied current are shown in (a) and (b), respectively. The graphs indicate insulator-to-metal transition when increasing the applied current from 12 mA to 14 mA, resulting in phase shifts ranging from 55° to 60° within 80-100 GHz frequency range.
